# Targeting of polycombs to DNA in EMT

**DOI:** 10.18632/oncotarget.20211

**Published:** 2017-08-12

**Authors:** Cecilia Battistelli, Marco Tripodi, Carla Cicchini

**Affiliations:** Department of Cellular Biotechnologies and Haematology, Istituto Pasteur-Fondazione Cenci Bolognetti, Sapienza University of Rome, Rome, Italy

**Keywords:** HOTAIR, Snail, EZH2, metastasis

Epithelial cells may respond to instructive signals and profoundly change the expression profile in a transdifferentiation process known as Epithelial-to-Mesenchymal Transition (EMT). Cells undergoing EMT progressively lose cell-cell adhesion and epithelial markers (primarily the E-cadherin) and rearrange apical-basal polarity and cytoskeleton to acquire mesenchymal markers and migratory capacity [1]. The plasticity of EMT is further underlined by the ability of transitional cells to undergo a reverse Mesenchymal-to-Epithelial Transition (MET) in secondary sites and changed cellular environment. All these features confer to EMT-MET dynamics a key role in developmental and physiological processes (such as organogenesis, tissue homeostasis, wound healing, regeneration, stem cell plasticity) as well as in fibrosis, tumor growth and, particularly, tumor metastasis, for which it represents a rate-limiting step [2].

EMT-MET balance implies a strict regulation of transcription. With respect to transcriptional factors, repressors of the Snail, ZEB and Twist families, and in particular the member Snail1 (Snail), act as “masters”, sufficient to trigger and guide the reprogramming. Furthermore, recent evidence demonstrated that epigenetic mechanisms are mandatory in EMT-MET dynamics and correlated Enhancer of Zeste Homolog 2 (EZH2), the Polycomb Repressive Complex 2 (PRC2) catalytic member, to Snail-mediated repression [3].

Conceivably, a master factor has the capacity to organize a molecular platform that profoundly impacts the chromatin state by i) recruiting chromatin modifiers to specific genes (thus triggering or impeding target genes transcriptional competency) and/or ii) allowing the enrolment of other transcriptional factors and/or co-regulators. Molecular mechanisms linking master factors and the main writers of chromatin repressive marks are still largely unknown.

In addition, molecular mechanisms by which Polycomb proteins interact with genomic targets are still debated in mammals (differently from what is found in Drosophila, in which DNA Polycomb Response Elements (PREs) are involved) where Polycomb members could be recruited to each gene through interactions with sequence-specific DNA-binding factors [4].

Notably, lncRNA HOTAIR was found to bind to PRC2, targeting its H3K27-mediated methylation to discrete regions of the genome [5]. While the PRC2-RNA interactions have been questioned as promiscuous in *in vitro* studies [6], *in vivo* HOTAIR is an excellent predictor of metastasis being able to induce a genome-wide PRC2 retargeting in epithelial tumors.

This evidence still leaves different possible mechanisms open (e.g. how is HOTAIR/EZH2 complex recruitedŒ The lncRNA directly promotes targeting or it modulates functionŒ).

In Battistelli et al. [7], different working hypotheses of how EZH2 gets to its genomic targets have been reconciled: provided evidence demonstrates as a DNA binding transcriptional factor can recruit to specific sites a general chromatin modifier by means of a long non-coding RNA, that is required to bridge the two proteins in a functional complex. This, in turn, allows the coordinated regulation of a broad repertoire of genes in reprogramming phenomena. At the same time, a new paradigm of function for a lncRNA has been highlighted: a lncRNA conveys to specific sites a general chromatin modifier through a DNA-binding transcriptional factor.

**Figure 1:**
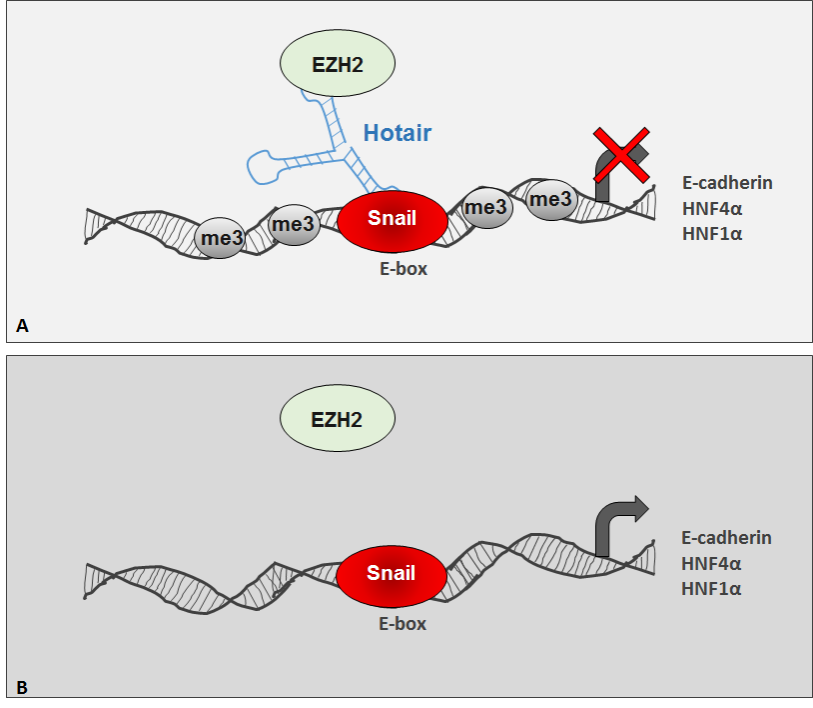
The Snail functional activity requires HOTAIR-mediated PRC2 member EZH2 recruitment Schematic model of Snail-mediated outcome in EZH2 recruitment for gene repression in cells expressing **A.** or not **B.** HOTAIR.

Battistelli and Coworkers demonstrated, specifically in hepatocyte cells, that the EMT master repressor Snail directs EZH2 activity to specific genes through the enrolment of the lncRNA HOTAIR (Figure 1). Functional *in vivo* analysis highlighted that the Snail repressive activity in EMT depends on the formation of this Snail/HOTAIR/EZH2 complex. Moreover, the HOTAIR-mediated recruitment of EZH2 by Snail has been proven necessary for repression of genes with a pivotal function in epithelial and hepatic morphogenesis differentiation (including E-cadherin and the epithelial master factor HNF4α). Thus, since HOTAIR bridging role is necessary for the assembly of a functional SNAIL/HOTAIR/EZH2 complex on chromatin, HOTAIR has an epistatic role on Snail function in EMT.

The understanding of basic molecular processes underlying Snail-mediated EMT holds promise for the development of novel therapeutic targets in carcinoma progression. In particular, taking into account that effective anti-metastasis approaches are still lacking, the comprehension of the role of HOTAIR paves the way for the design of strategies counteracting its dominant influence in metastasis.
